# Lineage Selection and the Maintenance of Sex

**DOI:** 10.1371/journal.pone.0066906

**Published:** 2013-06-18

**Authors:** Damien M. de Vienne, Tatiana Giraud, Pierre-Henri Gouyon

**Affiliations:** 1 Bionformatics and Genomics Programme, Center for Genomic Regulation (CRG), Barcelona, Spain; 2 Bionformatics and Genomics Programme, University Pompeu Fabra (UPF), Barcelona, Spain; 3 Ecologie, Systématique et Evolution, CNRS UMR 8079, Orsay, France; 4 Ecologie, Systématique et Evolution, Université Paris-Sud, Orsay, France; 5 Département Systématique et Evolution, Origine, Structure, Evolution de la Biodiversité, UMR 7205 CNRS-MNHN, Muséum National d'Histoire Naturelle, Paris, France; Fordham University, United States of America

## Abstract

Sex predominates in eukaryotes, despite its short-term disadvantage when compared to asexuality. Myriad models have suggested that short-term advantages of sex may be sufficient to counterbalance its twofold costs. However, despite decades of experimental work seeking such evidence, no evolutionary mechanism has yet achieved broad recognition as explanation for the maintenance of sex. We explore here, through lineage-selection models, the conditions favouring the maintenance of sex. In the first model, we allowed the rate of transition to asexuality to evolve, to determine whether lineage selection favoured species with the strongest constraints preventing the loss of sex. In the second model, we simulated more explicitly the mechanisms underlying the higher extinction rates of asexual lineages than of their sexual counterparts. We linked extinction rates to the ecological and/or genetic features of lineages, thereby providing a formalisation of the only figure included in Darwin's “The origin of species”. Our results reinforce the view that the long-term advantages of sex and lineage selection may provide the most satisfactory explanations for the maintenance of sex in eukaryotes, which is still poorly recognized, and provide figures and a simulation website for training and educational purposes. Short-term benefits may play a role, but it is also essential to take into account the selection of lineages for a thorough understanding of the maintenance of sex.

## Introduction

Some traits are considered to be “evolutionary dead ends”, initially developing due to short-term selective advantages, but resulting in lower rates of speciation or high rates of extinction in the long term, resulting in a species level selection [1], [2], [3]. Such traits illustrate the conflict between selection levels for traits that are advantageous within lineages but selected against between lineages. Species selection, for example, has been shown to maintain self-incompatibility in plants: the short-term advantages of self-fertilising individuals are offset by lineage selection, because only outcrossing species can be maintained in the long term [4]. Another example is provided by sociality in spiders, which has short-term benefits, due to cooperation, that are counterbalanced by long-term disadvantages associated with inbreeding in small cooperative units [5]. Specialisation in parasites may constitute another evolutionary dead ends, as high resource-specificity is advantageous in the short term but places lineages at greater risk of extinction, with a lower probability of diversification than more generalist lineages [6]. Similarly, body size in mammals provides short-term benefits due to the effects of ecological dominance, but increases the risk of species extinction [7].

Parthenogenesis is probably also an evolutionary dead end [8], [9], [10], with short-term demographic advantages for asexual organisms, but without the long-term advantages of recombination. Indeed, sex is costly in the short term, placing sexual lineages at a two-fold demographic disadvantage with respect to clonal lineages, but it probably has long-term advantages, due to recombination. The “paradox of sex” therefore concerns the predominance of sex as the principal mode of reproduction in eukaryotes despite its two-fold short-term disadvantages. Parthogenetic females can invade populations well before the long-term benefits of recombination (DNA repair, more rapid adaptation) can come into effect, and recombination disrupts beneficial combinations of alleles generated by past selection [11] and increases intra- and intergenomic conflict [12]. The paradox of sex thus remains a challenging question in evolutionary biology even after more than 40 years of research on this topic (see the first formalisation of the problem by Maynard Smith [13]).

The two-fold short-term advantage of asexuality led many researchers to search for counterbalancing two-fold short-term advantages of sex (for reviews, see [14], [15], [16]). Myriad models exist and many suggest that short-term advantages may be sufficiently large to account for the maintenance of sex: adaptation is beneficial in the short term for dealing with parasite co-evolution (the “red queen” hypothesis), or heterogeneous or rapidly changing environments, for purging deleterious mutations, for generating recombinants that are favoured in small populations in which genetic drift induces linkage disequilibrium [14], or for combinations of these reasons [16]. Some of these mechanisms have been demonstrated to act in a few model species [17]. However, the question remains unresolved as no one short-term mechanism is widely accepted as accounting for the maintenance of sex. Indeed, a careful examination of real cases reveals the situation to be complex: the mechanisms accounting for the maintenance of sex often appear to be specific to particular lineages and not widely applicable [8], [18]. It seems unlikely that sex is maintained by such a diversity of short-term causes. Instead, these findings suggest that selection may operate at a higher level, favouring lineages with constraints preventing the easy loss of sexual reproduction.

Indeed, sex appears to be essential in the short term, for diverse functions unrelated to recombination in various organisms [8], [18], [19]. For example, sex is often associated with the production of resistance or dispersal structures, particularly in plants and fungi (seeds, pollen and spores for long-range dispersal). In aphids, rotifers, plants and fungi, for example, eggs, seeds or spores provide resistance to harsh conditions, such as very cold winters or dry periods [20]. Such a strong link between sex and another important function results in serious short-term consequences of any loss of sex, due to the associated loss of resistance or dispersal structures. Other examples of strong links between sex and other important functions include fungal mechanisms for controlling transposable elements, the elimination of viruses or rejuvenation to overcome senescence, all occurring exclusively during meiosis [21], [22], [23]. It appears that species remain sexual because they cannot do otherwise, either because they cannot reproduce in the absence of sex (as in mammals, in which parthenogenetic eggs cannot develop correctly due to epigenetic mechanisms [24], and in *Drosophila*, in which parthenogenetic females are not very fertile [25], or because sex is associated with another, essential function (such as winter survival in aphids [20]). Finally, the current predominance of sexual species may reflect the constraint of the strong link between sex and survival structures. The species we see today are essentially those that could not afford to lose sexual reproduction. All the species that were able to avoid sex would have done so, but would subsequently have disappeared because they evolved too slowly in the long term. This hypothesis is consistent with selection at the lineage level [26], *i.e.*, with the selection of lineages unable to lose sex because this mode of reproduction is strongly linked to other essential functions.

The selection of sex at lineage level has been little investigated, despite widespread recognition of the long-term benefits of sex, which can purge deleterious mutations from the population and mediate adaptation to changing environments [27]. Indeed, all the mechanisms identified as potentially conferring short-term advantages of sex (e.g. DNA repair, evolution in response to changing environments) may even be more effective at providing long-term advantages. Indeed it seems very unlikely that these mechanisms can act fast enough to prevent the extinction of sexuals that is predicted to occur in very short periods of time: a single asexual appearing in a population of 1 million sexual individuals will invade in only 20 generations. Maynard Smith [13], in his first formalisation of the two-fold cost of sex, actually discussed the lineage selection hypothesis in depth and did not refute the underlying rationale. This was also argued as early as 1930 by Fisher [11]. Nunney [9] developed a lineage-selection model for the maintenance of sex, in which asexual lineages arose regularly (short-term advantage), but had higher extinction rates than their sexual counterparts (long-term disadvantage). Under these conditions, sex could be maintained because interlineage selection favoured species with a lower probability of becoming asexual. Lineage selection may therefore be the key element accounting for the maintenance of sex despite its short-term disadvantages (see also [28], [29], [30], [31], [32], [33]).

Nunney's work has, however, been largely neglected, and there has been little, if any, further exploration or discussion of the idea that lineage selection may account for the maintenance of sex. We therefore explore here, in more detail, lineage selection models accounting for the maintenance of sex. We propose two different models. In the first one we start with a single sexual lineage that may split, become extinct or become asexual at each time step (each generation). The fate of lineages is determined exclusively by extinction, speciation and transition rates. Our simulation is thus more illustrative than Nunney's model, as it represents a branching process similar to that acting in nature. Further, we let the transition rate change during the course of evolution. In our model, new lineages have a transition rate slightly different from that of their parents (randomly sampled from a normal distribution), so lineage selection can act on this trait. We thereby investigated whether lineage selection favoured lower rates of transition to asexuality (e.g. lineages in which a trait important for survival is linked to sexuality, making it difficult for these species to lose sexual reproduction).

The second model attempts to explore the reasons for the higher rates of extinction in asexual than in sexual lineages. It can thus be seen as a submodel of the first one, because it gives a justification for the values of parameters used in the first model. This second model is inspired directly from the single figure included by Darwin in “The origin of species” (1859, Chapter 4, [34]). At first glance, this diagram resembles a typical phylogenetic tree, with lines representing branches and tips representing species. However, in drawing this diagram, Darwin intended to give a meaning to the horizontal axis, with time represented on the vertical axis. Darwin explains this horizontal axis as follows:

*“[...] But as a general rule, the more diversified in structure the descendants from any one species can be rendered, the more places they will be enabled to seize on, and the more their modified progeny will be increased”*.

In Darwin's diagram, this statement is illustrated by lineages distant from each other along the horizontal axis being more likely to persist and to split. Conversely, lineages lying closer to each other along the horizontal axis are more likely to become extinct. Darwin does not discuss the level of these lineages, whether they correspond to isolated populations, species, genera or families. This diagram can thus be applied to any level of integration, so long as the lineages are isolated from each other. In modern terms, selection can act on any entity that can reproduce its kind, whether it is a genome, a family (kin selection) or any other taxon. Such process linking “distance” between lineages at a given time and divergence (or extinction) has been formalised and simulated in some models of speciation [35], [36]. We believe that such formalisation is useful in the context of lineage selection for the maintenance of sex. Indeed, when considering the issue of the maintenance of sex, asexual lineages may often be seen as less “diversified in structure” than sexual lineages because of the absence of recombination. Consequently, on the horizontal axis imagined by Darwin, sister asexual lineages are likely to most often lie closer together than sexual lineages. The higher rate of extinction for asexual lineages may therefore be explained by interlineage competition. Simulating the evolution of sexual and asexual lineages in this context also provides a meaningful graphical representation of the events occurring during the course of evolution. Phylogenetic trees are generally constructed to include only living species, with extinct lineages (which may correspond to the majority), not represented. However, the shape of such trees is undoubtedly largely determined by other lineages that were present in the past but are now extinct.

Here, we address the following questions: (1) Can the transition rate to asexuality evolve towards lower values by lineage-selection? (2) Can the specificities of sexual vs. asexual lineages in ecological competition influence the probability of maintenance of sex by lineage selection? Our aim was also to provide representations of the evolutionary history of sexual and asexual lineages for training and educational purposes.

## Materials and Methods

### Model without competition

**Figure 1:**
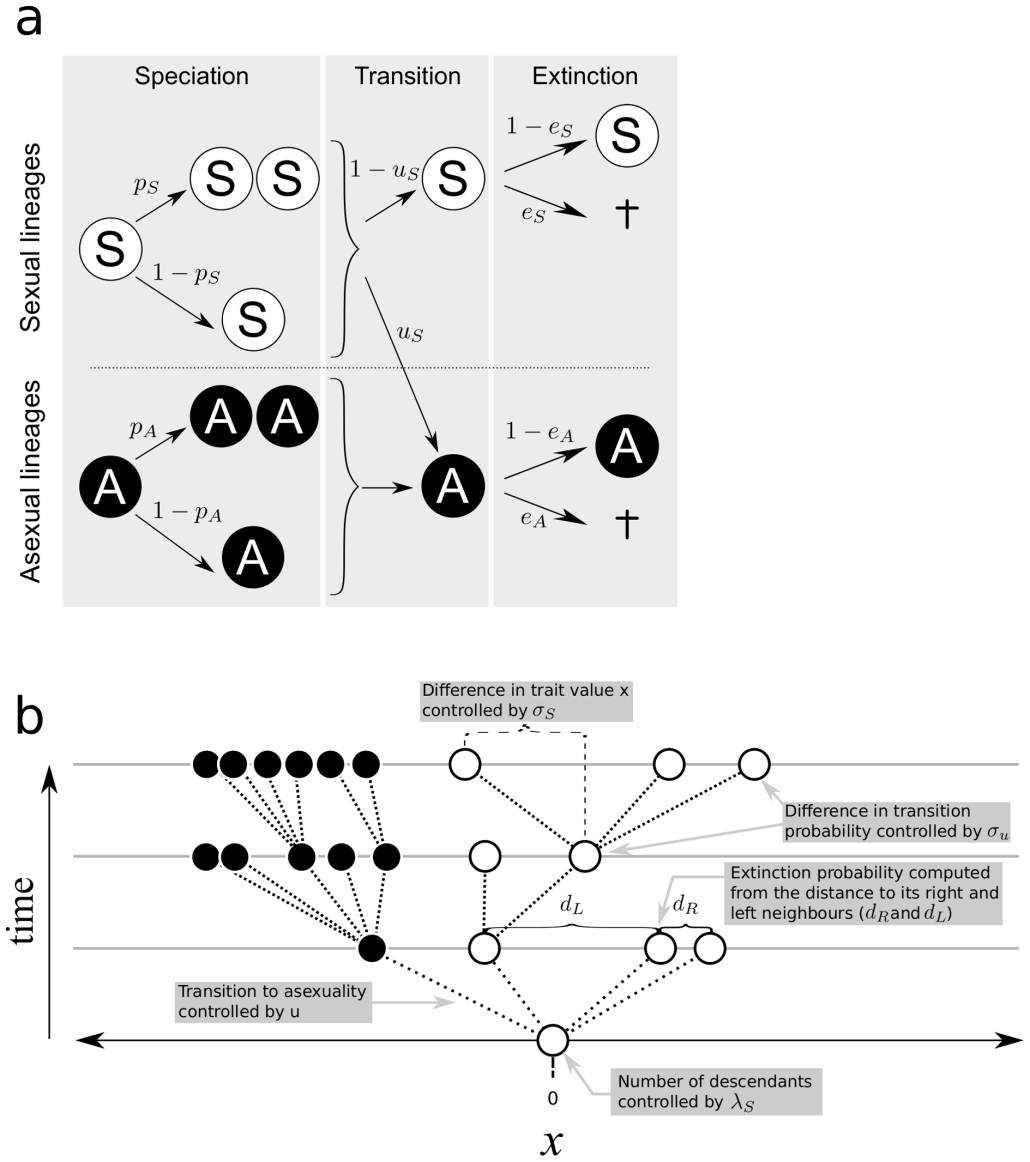
Schematic representation of the models developed here for exploring the maintenance of sexual reproduction. **a**: Model without competition. *p_S_* and *p_A_* are the speciation probabilities of sexual and asexual lineages, respectively, *e_S_* and *e_A_* are extinction probabilities for sexual and asexual lineages, respectively, and *u_S_* is the probability of transition from sexual to asexual states. While *p_S_*, *p_A_*, *e_S_* and *e_A_* are fixed over generations, the transition rate *uS* can evolve. **b**: Model with competition. The parameters are explained on the figure. For the two models, all simulations start with a single sexual lineage.

The first model includes a limited number of parameters. Simulations represent a branching process, with a single lineage at the beginning (as opposed to the constant number of species in Nunney's model) and transition rates evolve and are therefore potentially subject to selection. Mutations can change the rate of transition to asexuality at each step, in contrast to Nunney's model. The transition rate to asexuality reflects the cost of sex: it represents the probability that a parthenogenic female appears within the species and rapidly invades the sexual populations. Figure 1a provides a schematic representation of the model. At each generation, a lineage may speciate (with a probability *p_S_* for sexual species and *p_A_* for asexual species) or not speciate (with probabilities 1−*p_S_* and 1−*p_A_*, respectively). Sexual lineages may thus be transformed into asexual species, with a probability *u_S_*, or may remain sexual, with a probability (1−*u_S_*). Asexual lineages cannot become sexual again, so the rate of transition from asexuality to sexuality is 0. We assume that sex is too complex to be restored after this function has been lost. Lineages may become extinct with a probability *e_S_* for sexual lineages and *e_A_* for asexual lineages, or they may persist, with a probability (1−*e_S_*) for sexual lineages and (1−*e_A_*) for asexual lineages.

The transition rate 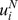
of each lineage *i* at each generation *N* is calculated from the transition rate 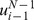
of the parent of lineage *i* (*i*−1) in the previous generation (*N*−1) as follows:
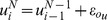
where 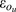
is sampled from a normal distribution with a mean of 0 and a standard deviation of *σ_u_*. There is no direct selection at the individual level on this transition rate.

Simulations were run for 2000 generations, with various values for *p_A_*, *p_S_*, *e_A_*, *e_S_*, *σ_u_* and *u_init_*, where *u_init_* is the initial rate of transition assigned to the first sexual species at the start of the simulation.

Some relationships and constraints were assigned to the parameters presented above. The speciation rate of asexual lineages was assumed to be twice that of sexual lineages [37]:
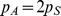



Speciation is assumed to be easier in asexual lineages because they do not experience recombination that is predicted to prevent speciation by breaking the adaptive combination of alleles [38]. The extinction rate for asexual lineages was assumed to be higher than that of sexual lineages:
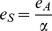
where *α* indicates the magnitude of the difference between the extinction rates of sexual and asexual lineages. We performed simulations for *α* = 2 and *α* = 5.

Finally, we restricted the analysis to situations in which the speciation rates of sexual lineages were higher than their extinction rates (*p_S_* > *e_S_*), to ensure that not all lineages became extinct before the end of the simulation.

Furthermore, for lineages reaching a transition rate of zero (no possibility of transition to asexuality), we assigned the same value to its descendants, which were thus also unable to give rise to asexual descendants. This choice was based on the observation that sexual species that appear today to have a zero probability to lose sex due to strong constraints will probably not be able to lose sex on the long term either. Humans for example will very unlikely become ever asexual naturally.

### Model with competition

This second model was inspired from Darwin's diagram in “The Origin of Species” and is presented in Figure 1b. There are three distinct phases at each generation.

#### 1. Competition between lineages

The outcome of this competition depends on the similarity of the lineages in terms of their ecological traits, as indicated by a value on an axis, regardless of the sexual state of the lineages. Basically, if two lineages have very similar values for these traits, they have a higher probability of extinction. This illustrates the classical niche competition hypothesis, according to which, two species with similar niches cannot coexist.

#### 2. Generation of new lineages

The persisting lineages can give rise to descendant lineages at the next generation, with a probability corresponding to their speciation rate. The number of descendant lineages is sampled from a Poisson distribution (see the parameters section).

#### 3. Assignment of traits to the new lineages

An ecological trait value is assigned to each new lineage, based on both the value of the parental lineage and some random evolution of this trait.

### Parameters

#### Competition between lineages

Competition between lineages controls the probability of extinction of each lineage, in each generation. It is dependent on the distance between the ecological trait value of the lineage and those of its neighbours to the left and to the right on the horizontal axis. The equation giving the probability of a lineage becoming extinct is:
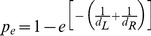
(1)where *d_L_* and *d_R_* are the distances to the nearest neighbours of the trait considered, to the left and to the right, respectively, on the horizontal axis (*i.e.* the difference in trait value). If the lineage has no neighbour on the right or left (*i.e.* if it is the last lineage on the left or on the right of the axis), the corresponding fraction *1/d* is removed.

#### Generation of new lineages

The number of lineages descending from each lineage is chosen randomly from a Poisson distribution with parameter *λ*, which is different for sexual (*λ_S_*) and asexual (*λ_A_*) lineages. We set *λ_S_* to *λ_A_*/2, to reflect the demographic advantage of asexual lineages, which are therefore more likely to generate larger numbers of daughter lineages, due in particular to the reproductive isolation induced by the absence of sex. A larger number of separate lineages would therefore be expected than for sexual lineages, in which gene flow occurs.

#### Traits of the new lineages

The trait value is 0 at the beginning of the simulation. This trait is called *x* and is calculated as follows for each lineage *i* at each generation *N*:
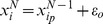
(2)where *i_P_* represents the parent of lineage *i* and *ε_σ_* is sampled from a normal distribution with a mean of 0 and a standard deviation of *σ*. The value of *σ* differs for sexual (*σ_S_*) and asexual (*σ_A_*) lineages. The long-term advantage of sex is included in the model by assuming that *σ_S_* > *σ_A_*, implying that sexual lineages can evolve more rapidly to new environments.

#### Mode of reproduction of lineages and evolution of the transition rate *u*

An initial transition rate *u_init_* is set at the beginning of the simulation for the first (ancestral) sexual linage. Each time a new lineage is formed, the probability of its becoming asexual is *p_S→A_*  = *u* and the probability of its remaining sexual is *p_S→S_*  = 1 – *u*. The probability of a reverse transition *p_A→S_* is set to 0 so that *p_A→A_*  = 1. The transition rate *u* evolves at each speciation event. It is calculated as follows for each lineage *i* at each generation *N*, as long as the lineage remains sexual:
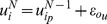
(3)where *i_P_* represents the parent of lineage *i* and 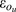
is sampled from a normal distribution with a mean of 0 and a standard deviation of *σ_u_*.

As for the first model, for lineages reaching a transition rate of zero (no possibility of transition to asexuality), we assigned the same value to its descendants, which were thus also unable to give rise to asexual descendants.

### Simulations

Refer to Figure 1 and Table 1 for a description of the parameters.

**Table 1:** List and description of the parameters used in the two models.

	Parameters	Description
Model without competition	*p_A_*	Speciation probability of asexual lineages
	*p_S_*	Speciation probability of sexual lineages
	*e_A_*	Extinction probability of asexual lineages
	*e_S_*	Extinction probability of sexual lineages
	*u_init_*	Initial rate of transition from sexuality to asexuality, i.e. the transition rate assigned to the initial single sexual lineage at the beginning of simulation
	*σ_u_*	Parameter controlling the rate of change in the transition rate. The higher the value of this parameter, the greater the difference between each descendant lineage and its predecessor in terms of rate of transition to asexuality
	*α*	Parameter controlling the difference between sexual and asexual lineages in terms of extinction rate. Asexual lineages have an extinction probability α-times greater than that of sexual lineages.
Model with competition	*λ_A_*	Parameter controlling the number of descendants of asexual lineages at each generation. The number of descendants is sampled from a Poisson distribution with parameter *λ_A._*
	*λ_S_*	Parameter controlling the number of descendants of sexual lineages at each generation. The number of descendants is sampled from a Poisson distribution with parameter *λ_S_*
	*σ_A_*	Parameter defining how different in ecological traits (horizontal axis) an asexual lineage is from its predecessor.
	*σ_S_*	Parameter defining how different in ecological traits (horizontal axis) a sexual lineage is from its predecessor
	*p_e_*	Extinction probability of sexual and asexual lineages
	*x_i_*	Ecological trait value (co-ordinate on the horizontal axis) of lineage *i*
	*u_init_*	Initial rate of transition from sexuality to asexuality, i.e. the rate of transition assigned to the initial single sexual lineage at the start of the simulation
	*σ_u_*	Parameter controlling the rate of change in transition rate. The higher the value of this parameter, the greater the difference between each descendant lineage and its predecessor in terms of the rate of transition to asexuality

For the model without competition, we simulated 2000 generations for each set of parameters and carried out this operation 20 times. We performed simulations with *p_S_* set to 0.002, 0.003, 0.004 and 0.005 and *e_S_* set to 0.001, 0.002, 0.003 and 0.004, applying the constraint that *p_S_* had to be higher than *e_S_*. We tested values of *u_init_* and *σ_u_* between 0 and 10^−2^ (0, 10^−6^, 10^−4^, 10^−2^).

For the model with competition, the simulations were as follows: 500 generations, *σ_S_* fixed at 100 and *σ_A_* varied between 10 and 100 (10, 20, 30..., 100). We set *λ_S_* to 3 and *λ_A_* to 6 and we tested four different values for *u_init_* and *σ_u_*: 0, 10^−5^, 10^−3^ and 10^−1^. We carried out 10 simulations for each set of parameters.

## Results

For clarity, Figure 1 presents the two models developed here and Table 1 lists the parameters used in and their biological meaning.

### Model without competition

**Figure 2:**
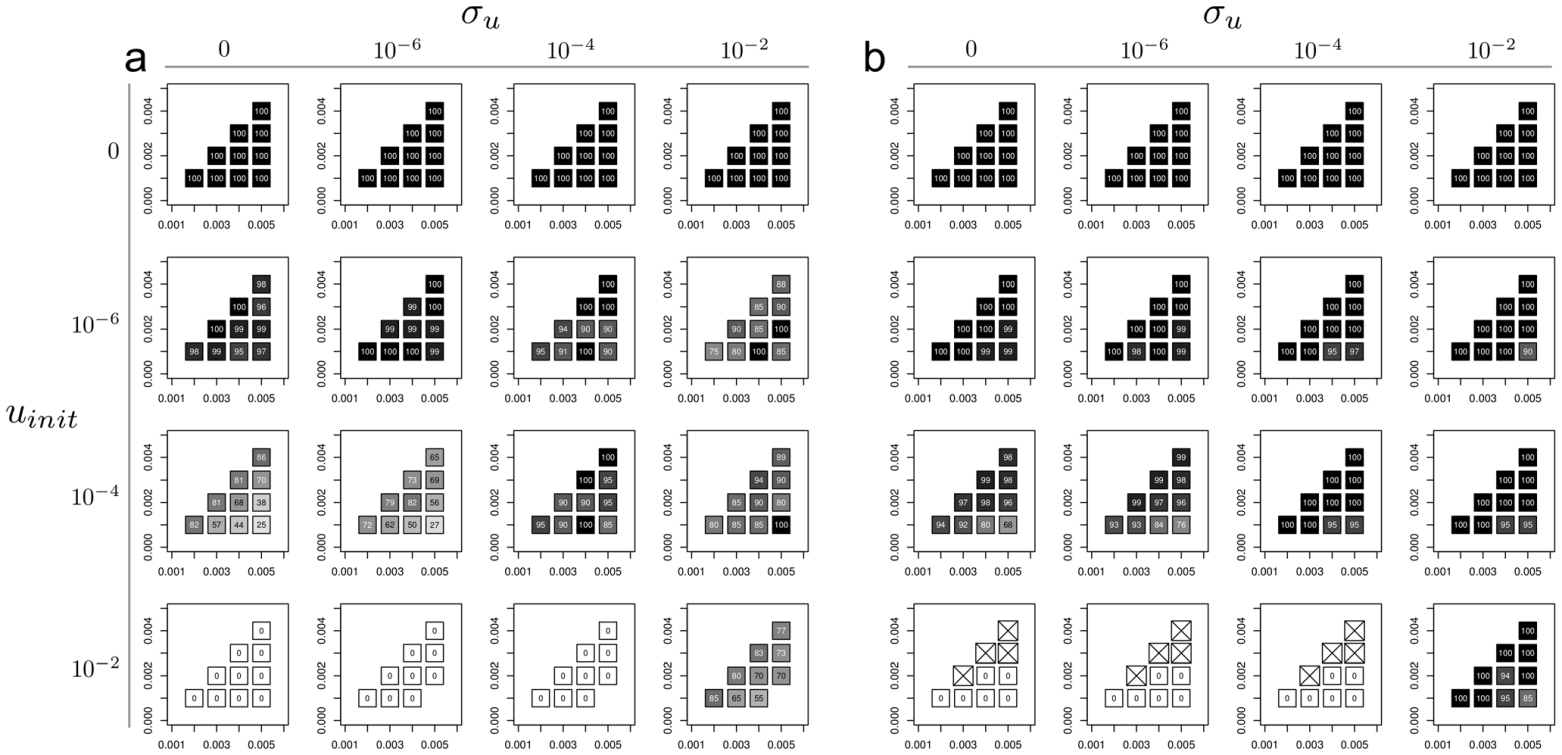
Proportion of sexual lineages at the end of simulations for the model without competition, for different values of the initial transition rate (*u_init_*, rows), the rate of change of this transition rate (*σ_u_*, columns), the speciation rate of sexual (*p_S_*, x-axis on each plot) and the extinction rate of sexual (*e_S_*, y-axis on each plot). Plots **a** and **b** differ by the value of *α*, a parameter that controls the difference between sexual and asexual lineages in terms of extinction rate (asexual lineages have an extinction probability α-times greater than that of sexual lineages). *α* = 2 in **a** and *α* = 5 in **b**. The shading represents the mean proportion of sexual lineages at the end of the runs (after 3000 generations, 50 repetitions). Cells with crosses (×) represent cases in which all simulations ended prematurely.

The results for the success of sexual and asexual lineages for various values of sexual lineage speciation (*p_S_*, *x*-axis) and extinction (*e_S_*, *y*-axis) rates, initial transition rate *u_init_* and the rate of change of the transition rate *σ_u_* are summarised in Figure 2. The percentage of sexual lineages at the end of the simulations (after 2000 generations) is given for *α* = 2 (asexual lineages twice as likely as sexual lineages to become extinct, Figure 2a) and *α* = 5 (asexual lineages five times more likely than sexual lineages to become extinct, Figure 2b).

When the initial transition rate is zero (*u_init_*  = 0, first row in Figure 2a and 2b), no asexual lineages are generated and the percentage of sexual lineages at the end of the simulations is 100%, because we do not allow lineages with a null transition rate to give rise to descendants with a non-null transition rate.

If the transition rate cannot evolve (*σ_u_*  = 0), the success of sexual lineages depends solely on the initial transition rate *u_init_* and the extinction and speciation rates for sexual lineages (and asexual lineages, indirectly). The effect of the initial transition rate on the success of sexual lineages is clear in conditions in which the difference in extinction rates between sexual and asexual populations is not too large (for α = 2, Figure 2a), because asexuality is not particularly advantageous in these conditions. An increase in the initial transition rate (from 0 to 10^−2^) seems to be associated with a decrease in the proportion of sexual lineages at the end of the simulation. This is expected, as an increase in the initial transition rate implies an increase in the probability of a sexual lineage giving rise to asexual descendants. The effect of the speciation and extinction rates of sexual lineages is also very clear when we consider the situation in which *α* = 2, *σ_u_*  = 0 and *u_init_*  = 10^−4^. When the speciation and extinction rates of sexual lineages are similar (0.002 and 0.001, 0.003 and 0.002, etc.), the percentage of sexual lineages at the end of the simulation is higher than in conditions in which the rate of speciation is higher than the rate of extinction. For example, for *α* = 2, *σ_u_*  = 0 and *u_init_*  = 10^−4^, only 25% of the lineages are sexual at the end of the simulation if the speciation rate of sexual lineages is 0.005 and their extinction rate is 0.001, versus 86% if sexual lineages have an extinction rate of 0.004.

**Figure 3:**
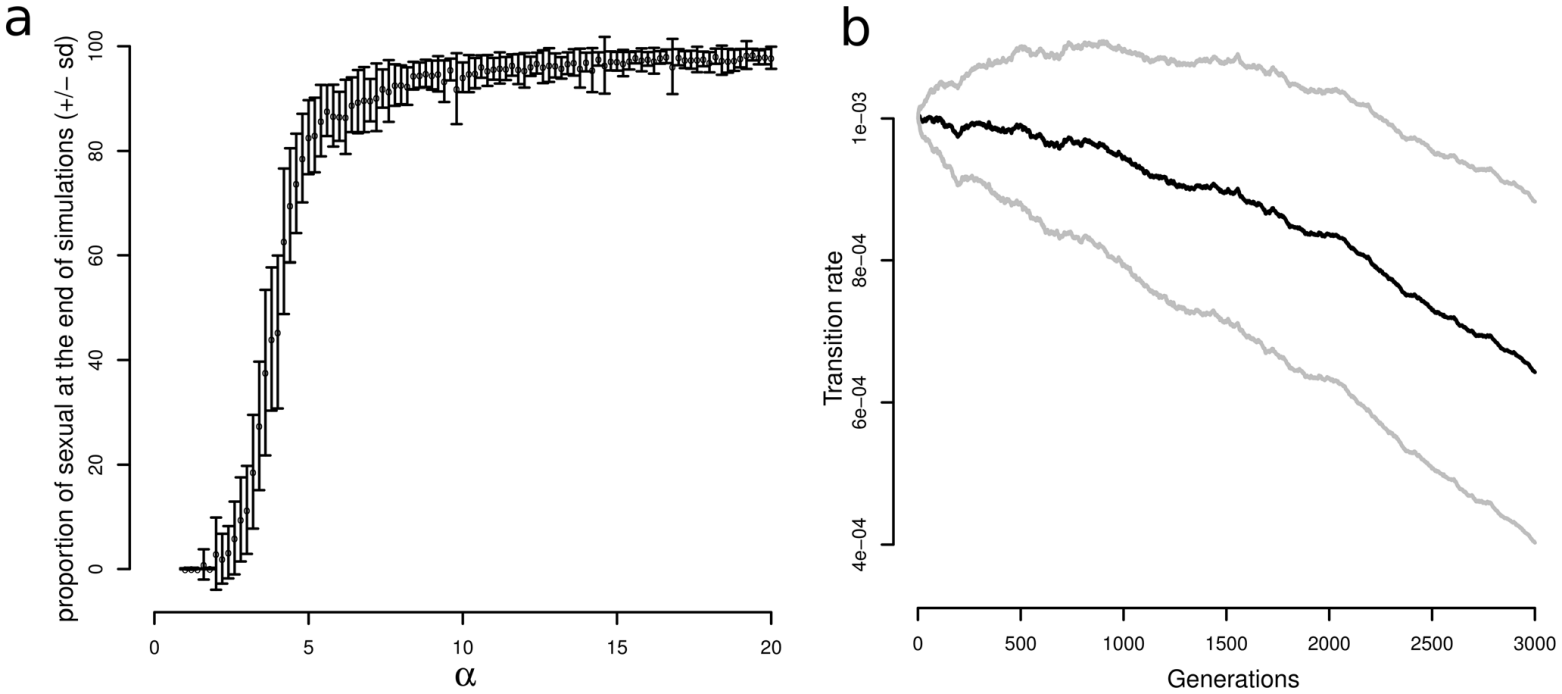
Effect of different parameters on the proportion of sexual lineages at the end of simulations (a) and the evolution of the transition rate of sexual (b) for the model without competition. **a**. Effect of increasing the difference in the rate of extinction between asexual and sexual lineages (controlled by the α parameter, see Table 1; x-axis) on the proportion of sexual lineages at the end of simulations. The vertical bars indicate the standard deviation around the mean. The parameters used are as follows: Extinction rate of sexual lineages: *e_S_*  = 0.002; Speciation rate of sexual lineages: *p_S_*  = 0.004; initial transition rate: *u_init_*  = 10^−3^; Rate of change in the rate of transition: *σ_u_*  = 10^−5^. **b**. Changes in transition rate over 3000 generations. Mean and SD were calculated for 50 repetitions for the same set of parameters as in **a**. and with α = 10.

The effect of *α* on the success of sexual lineages is difficult to determine from Figure 2, in which only two values were tested. We therefore performed simulations with fixed values for the extinction rate (*e_S_*  = 0.002) and the speciation rate (*p_S_*  = 0.004) of sexual lineages, and for an initial transition rate (*u_init_*) of 10^−3^ and a rate of change in transition rate (*σ_u_*) of 10^−5^. We varied the value of *α* from 1 to 20, by steps of 0.2. We performed 100 repetitions for each value of *α*, allowing the simulation to run for 3000 generations. A value of *α* = 4 indicates that the extinction rate is four times higher for asexual lineages than for sexual lineages. The mean percentage of sexual lineages at the end of simulations was found to be a function of the value of *α* (Figure 3a). Note that the initial conditions in this case are favourable to asexual lineages, as each sexual lineage at each generation has a probability of becoming asexual of 1/1000. When *α* is small, sexual lineages are completely eliminated and only asexual lineages remain at the end of the simulation, as expected with the chosen parameters. When *α* increases, the proportion of sexual lineages at the end of simulations increases; for values of *α* greater than 4, sexual lineages predominate at the end of the simulations.

The main conclusion from our analysis of this first model is that, even under conditions favouring the asexual forms and preventing the occurrence of new sexual lineages, sex can be maintained by lineage selection so long as the transition rate can change and/or the extinction rate of sexual lineages is much lower than that of asexual lineages. However, when the rate of extinction of asexual lineages is only twice that of sexual lineages (the rate of speciation of asexual lineages being twice that of sexual lineages), sex may be maintained if the initial transition rate is low or if the initial transition rate is high but can change.

We demonstrate the importance of changes in transition rate for the success of sexual lineages in Figure 3b, in which we show the mean transition rate for *α* = 10 over the 3000 generations. Transition rate seems to decrease over time, with lineages having too high a transition rate being transformed into asexual lineages that eventually become extinct due to their higher extinction rate.

### Model with competition

**Figure 4:**
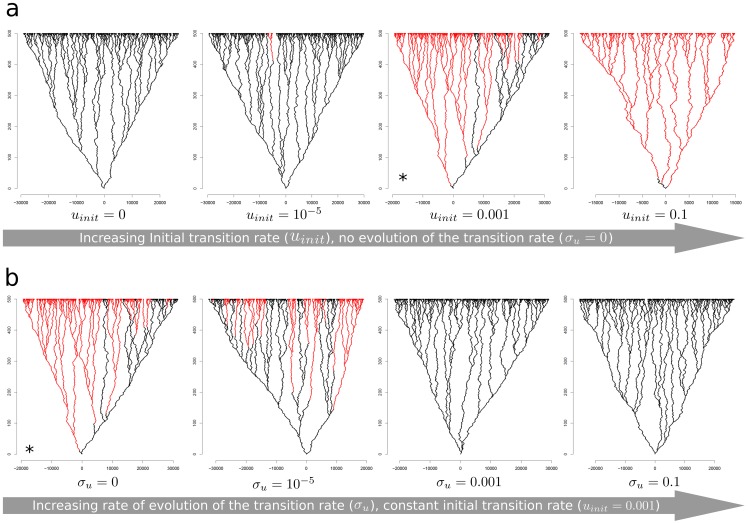
Effect of the initial transition rate (a) and of the rate of change in transition rate (b) on the sexual and asexual lineages formed during simulations, for the model with competition. Black lines represent sexual forms and red lines represent asexual forms. Extinct lineages are not represented. The tree with a star (*) in **a** is the same as the tree with a star (*) in **b** (same set of parameters).

This new model provides access to the same information as the previous one: percentage of sexual and asexual lineages at the end of simulations, and transition rate over the simulations. It also involves the recording and visualisation of additional relevant biological features, such as the timing of the transition events and the number of sexual and asexual forms present in the past. Graphical representations of the trees are also produced, in which each line links an ancestral form to its descendants (Figure 4).

#### Effect of the initial transition rate and its rate of change on the success of sexual lineages

**Figure 5:**
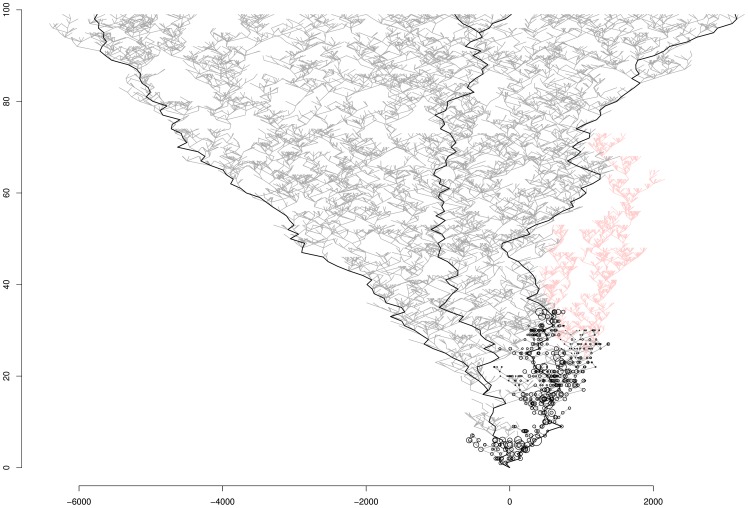
Illustration of lineage selection favouring sexual lineages with the model with competition. The circles represent the probability of transition from sexuality to asexuality. Black and grey lines represent sexual forms, and red lines represent asexual forms. Lineages without circles can no longer lose sex. After 100 generations, the lineages present are all sexual and have lost the ability to become asexual; all the asexual lineages have become extinct.

We first looked at the effect of different initial transition rates (*u_init_*) on the success of sexual forms with a fixed rate of transition (*σ_u_*  = 0) (Figure 4a and Figure 5). We set *σ_S_* to 100, *σ_A_* to 60, *λ_S_* to 3 and *λ_A_* ato 6. As expected, higher initial transition rates were associated with a higher proportion of asexual lineages (black lines in Figure 4a) than of sexual lineages (red lines in Figure 4a) at the end of the simulation. This is because, with a null or very low initial transition rate (*u_init_*  = 0 and *u_init_*  = 10^−5^, Figure 4a), very few if any asexual lineages emerge because the probability of a transition from sexual to asexual reproduction is too small to happen. By contrast, when the initial transition rate is high (*u_init_*  = 0.001 and *u_init_*  = 0.1), the probability of transition is high enough for such transitions to occur many times, and the asexual forms predominate after 300 generations.

If we begin with a set of parameters favourable to asexual forms (first tree in Figure 4b, identical to the 3^rd^ tree in Figure 4a), increasing the rate of change of the transition rate (*σ_u_*) appeared to increase the proportion of sexual lineages. Indeed, the proportion of sexual lineages at the end of simulations increased with increasing *σ_u_*. This may be because the sexual lineages lost the ability to become asexual rapidly, such that the asexual lineages did not have time to expand. Furthermore, as asexual lineages had a higher extinction rate (indirectly, because *σ_S_*  = 100 and *σ_A_*  = 60), sexual lineages eventually predominated, as such lineages tend to lose their ability to become asexual, whereas the asexual lineages that emerged at some point in the simulation eventually become extinct. As an illustration of this process, we generated a plot similar to those presented in Figure 4 but also showing all the extinct lineages (Figure 5). Moreover, for each sexual lineage, we plotted the probability of transition to asexuality (black circle in Figure 5). The lineages of the left branch rapidly lose their ability to become asexual. By contrast, those on the right branch retain this capacity until a transition occurs, giving rise to the asexual clade in Figure 5. However, the asexual clades become extinct after some generations, as they extend over only a small part of the horizontal axis. Thus after 100 generations for this simulation, only sexual lineages are present and these lineages can no longer give rise to asexual forms.

**Figure 6:**
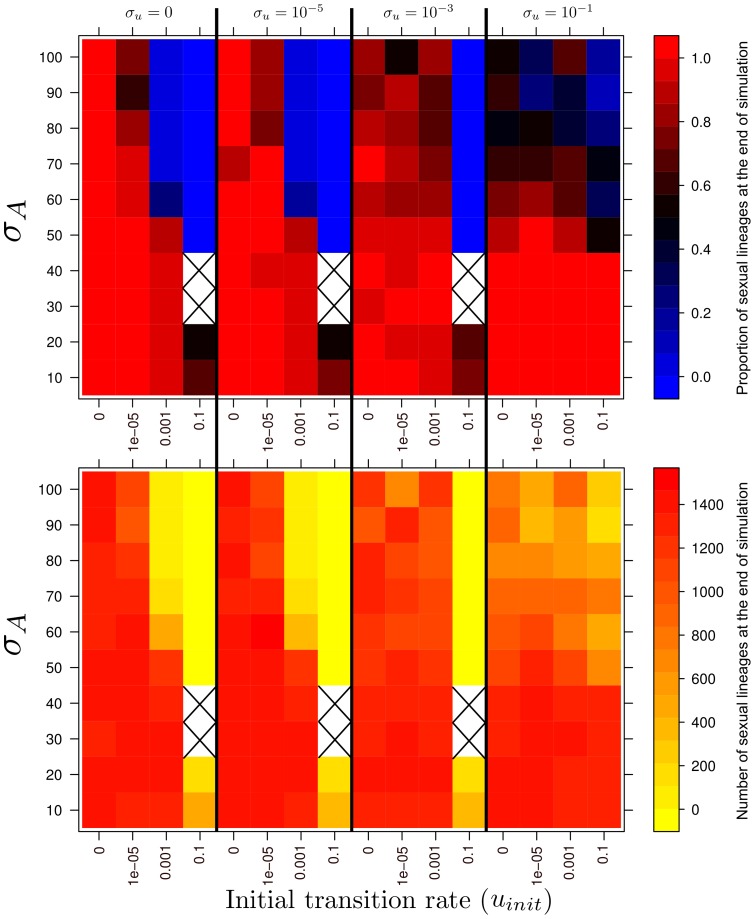
Heatmaps showing the mean proportion (top panel) and mean number (bottom panel) of sexual lineages at the end of simulations (500 generations) over 10 repetitions, for various values of initial transition rate (*u_init_*), rate of change in the transition rate (*σ_u_*) and the “exploratory ability” of asexuals on the horizontal axis (*σ_A_*). *σ_S_* is fixed at 100.

Figure 6 presents all the results obtained with this model in a more analytical way, in terms of (i) the proportion of sexual forms at the end of simulation and (ii) the absolute number of sexual forms at the end of simulation, as a function of the initial transition rate *u_init_*, the ability of asexual forms to explore the horizontal axis (*σ_A_*), and the rate of change of the transition rate (*σ_u_*).

Overall, the proportion of sexual forms present at the end of simulations decreases with increasing initial transition rate. However, changes in the transition rate across generations favour sexual lineages. Indeed, even under conditions largely detrimental to sexual lineages (*u_init_* = 0.001, *σ_A_*  = 60, 30% of the lineages are sexual at the end of simulation), increases in the rate of transition may lead to a final proportion of sexual forms at the end of the simulation of more than 80% (for *σ_u_*  = 0.001, Figure 6).

## Discussion

Our study reinforces the view, still too poorly recognized, that the maintenance of sex can be explained by lineage selection, based on long-term advantages. Our model indeed allowed the maintenance of sexual lineages without the need to assume the existence of short-term advantages. We show that lineage selection can favour the gradual evolution of low rates of transition to asexuality and that the specificities of ecological competition between asexual and sexual lineages influence the possibility of maintenance of sex. Indeed, the greater difference in the ability of sexuals versus asexuals to diversify and occupy more diverse ecological niches (i.e. the greater *σ_S_/σ_A_*), the easier it was for sex to be maintained. This is intuitive but had not been explored before, and corresponds to Darwin' unique figure in “The Origin of Species”. Empirical studies have in fact shown that asexual and sexual closely related populations or species differ in ecological niches, with a wider niche for sexuals [e.g. 39,40,41,42]). These studies however remain scarce while our simulations indicate that it could be an important feature to explore.

The finding that lineage selection can favour the evolution of low rates of transition to asexuality results from lineages with high rates of transition to asexuality going extinct rapidly due to the long-term costs of a lack of recombination. This may account for most of the short-term advantages of sex being associated with sex itself but not with recombination: the lineages that are maintained in the long term are those that cannot afford to lose sex (i.e. those with a low rate of transition to asexuality). Many examples of short-term advantages of sex unrelated to recombination making it difficult for an organism to lose sexual reproductive function have been described, including the formation of dispersal structures, eggs or resistant spores [23], the control of transposable elements [21], virus elimination [22], rejuvenation in a context of senescence [43], imprinting in mammals [18], [24], lower fitness of clonal females [18] and selection arena [19], [44].

We do not claim that there are never short-term advantages to recombination in particular conditions or lineages, and we agree with West *et al.* [16] and Otto [14] that a pluralist approach to sex will provide more insight into the mechanisms maintaining sex than individual approaches considered in isolation. However, although the putative short-term advantages of recombination have been studied in detail, both experimentally and theoretically, long-term advantages and lineage selection seem to constitute a largely neglected, but fundamental aspect of the question of the maintenance of sex in eukaryotes (but see [8], [9], [18]). According to the lineage selection hypothesis, sex is selected principally at the species level, favoring the selection of lineages that cannot lose the ability to reproduce sexually, for whatever reason. The idea is that all the species that were able to become asexual did so, but that most became extinct in the long term. As a result, the species we observed today are mostly sexual because they were unable to lose sexual reproduction as it was linked to another function that was essential in the short term.

Three main lines of evidence can been put forward to support the idea that lineage selection is responsible for the maintenance of sex. First, most asexual lineages appear to be recent, as shown by their “twiggy” pattern in phylogenetic trees [45]. This implies that most of the asexual lineages that emerged in the more distant past have become extinct, indicating that this trait is subject to rapid species-level selection. Second, if there were short-term advantages of sex that were strong enough to counterbalance the two-fold short-term disadvantages from which they suffer at each generation, asexual lineages would be unlikely to persist for more than a dozen generations. Indeed, one would need a two-fold advantage of sex over parthenogenesis at each generation to counterbalance the two-fold cost of males at each generation. For 20 generations, sexual lineages need to counterbalance a cost of 10^6^. This means that, to be maintained at the same frequency across 20 generations, asexual lineages need to have short term disadvantages by the same factor 10^6^, due for instance to maladaptation to parasites or the accumulation of deleterious mutations. Asexual lineages are recent, but have nonetheless existed for many thousands of generations [46]. The asexual individuals alive today would therefore be expected to be less fit than their ancestors by a factor of billions, which does not appear to be compatible with their persistence. Extant asexual species might be considered to be very unusual, in that they do not appear to suffer any ill effects of the loss of sex, either because they do not carry any parasites or because they have acquired very efficient DNA repair mechanisms. However, no such unusual features have been found in most asexual species (except in the very peculiar bdelloid rotifers, [47]). Third, the diversity of traits/features put forward as accounting for the maintenance of sex in eukaryotes suggests that selection acts at a higher level, to retain an essential function linked to sex, through the selection of a low rate of transition from sexuality to asexuality. Indeed, it appears not parsimonious that a phenomenon as general as sex could be maintained by independent and different causes.

The hypothesis that lineage selection can account for the maintenance of sex could be tested by investigating whether asexual lineages indeed have higher extinction rates and are younger than sexual lineages. Indeed, if clonal lineages experience higher extinction rates, then there will be an automatic sorting (i.e. a selection) of lineages. If there is variation in the rate of transition to asexuality, this sorting will result in an over-representation of lineages with a low probability of losing sex. It is widely accepted that clonal lineages have higher extinction rates, but this hypothesis has been surprisingly little tested. Some recent studies have suggested that the hypothesis of a “twiggy pattern” of asexual lineages should be tested against a hypothesis of neutral processes [48], [49].

The idea that recombination has huge long-term benefits is so widely accepted that ancient asexual lineages, such as the bdelloid rotifers, have been called “evolutionary scandals” [50] and many studies have tried to understand how they have survived for so long [51], [52]. However, even the bdelloid rotifers do not seem to be “scandalously ancient” [46]. Indeed the distribution of asexual lineage age follows a regular distribution, with the asexual taxa viewed as “scandalously” ancient merely lying at the upper end of this distribution [46]. This suggests that similar mechanisms may determine asexual lineage age across eukaryotic taxa.

Several major scientists with strong personalities have contributed to this debate. Stephen J. Gould made the point that the rejection of selection at species level was a firmly established feature in the ideas of most of the leading figures in evolutionary genetics of the late 20^th^ century [53]. Maynard-Smith did not rule out this possibility [13], but other scientists have displayed strong antipathy against processes of this type. Gould wrote [53]: “To say (as Dawkins, Williams, and other detractors often do) that species selection must be unimportant because such a process can't build organismal complexity reminds me of the cook who didn't like opera because singing couldn't boil water”. This surprising metaphor is very relevant here: species selection clearly cannot explain the origin of sex, but it could be useful for explaining its maintenance in situations of anisogamy. Understanding the structure of diversity in the tree of life and, particularly, of the existence of sex in living lineages, may well be required, not “to make the water boil” (that is to understand how *organismal complexity* was built), but rather to allow the diversity of selective processes at the different scales of the tree of life “to sing” (that is to understand how the *complexity of the tree of life* was built).

We are therefore unlikely to be able to develop a complete understanding of the maintenance of sex in eukaryotes if we do not consider all levels of selection. Indeed, one possible answer to the question “Why do aphids reproduce sexually?” would be “because they cannot produce resistant forms in any other way”. However, the most correct answer to the question “Why do most species reproduce sexually?” may be “because all the other species became extinct”. Both answers may be correct, but these two questions do not address the same level of selection. Ideally, if we wish to obtain a comprehensive understanding of the maintenance of sex in eukaryotes, we need to identify all the short-term causes in each species, together with the long-term forces in action. However, it is becoming clear that we do not necessarily need to know all the proximate causes, whereas ignoring even one final cause would greatly damage our overall understanding of the evolutionary problem.

Lineage selection can occur as soon as the different lineages become isolated from each other, such that lineages cannot be invaded by the individuals with short-term demographic advantages that have invaded other lineages. The finding that so many different proximal causes seem to be responsible for the maintenance of sex in different lineages strongly suggests that a higher level of selection is at work, consistent with the widely accepted view that recombination has long-term advantages.

### Availability

The C source code for the second model and a browser for exploring and visualizing all the trees generated with this model is available online at http://lineage-selection.cgenomics.org.
